# Piloting an Alternative Implementation Modality for a School-Based Child Sexual Abuse Prevention Curriculum

**DOI:** 10.3390/ijerph21020149

**Published:** 2024-01-29

**Authors:** Nusrat E Mozid, Rebeca N. Espinosa, Corinne Grayson, Oluwatumininu Falode, Yilei Yang, Christelle Glaudin, Kate Guastaferro

**Affiliations:** School of Global Public Health, New York University, New York, NY 10003, USAoof218@nyu.edu (O.F.); yy3193@nyu.edu (Y.Y.);

**Keywords:** child sexual abuse, implementation, prevention

## Abstract

Background: In the U.S., the most pervasive child sexual abuse (CSA) prevention strategy involves school-based prevention programs; however, the reach of these programs is limited due to implementation constraints, such as budgets or turnover. This is notable as standard delivery of often requires two facilitators in the classroom. Leveraging a natural experiment in the implementation of *Safe Touches*, the current study sought to explore the feasibility of implementation with a single facilitator using pre-recorded videos compared to the standard in-person delivery. Methods: A six-item CSA-related knowledge questionnaire was delivered to (N = 1480) second-graders post-workshop. An independent-samples *t*-test was used to compare the mean of CSA-related knowledge item responses for each delivery modality. Student-level data were paired with teacher evaluations and an interview with the facilitator. Results: Across workshops delivered in 25 schools, there was no significant difference in knowledge based on CSA-related questions by workshop modality. Teachers indicated the facilitators responded effectively to the children’s questions and comments in both delivery modalities. Input from the facilitator was positive. Conclusions: Triangulation of student knowledge, teacher input, and facilitator experience indicates the viability and feasibility of this implementation strategy for *Safe Touches*, and potentially other school-based CSA prevention programs. To ensure equitable access to the CSA prevention program, the empirical examination of, and investment in, alternative implementation options for school-based CSA preventive programs is encouraged.

## 1. Introduction

Child sexual abuse (CSA), a subtype of child maltreatment, includes any direct physical contact and/or noncontact sexual act toward a child under 18 years of age, in which there is no, or limited, capacity to provide true consent [1]. In the United States (U.S.), an estimated 1 in 5 girls and 1 in 20 boys under the age of 18 years are believed to be victims of CSA each year [2,3]. Children who have experienced CSA are prone to a variety of adverse biopsychosocial outcomes [4], including obesity, poor mental health, and substance use [5,6,7,8,9], contributing to a lifetime economic burden estimated to exceed USD9.3 billion [10]. The most prevalent, and cost-effective [11], primary prevention strategy in the U.S. involves universal school-based interventions, several of which have been shown to significantly increase children’s self-protection awareness and skills [12,13,14]. Some interventions have also been associated with facilitating disclosures of abuse, an encouraging and crucial step in prevention [15,16]. However, for the past two decades, more than 60,000 children are consistently determined to be victims of CSA by child protective service systems [17]. This suggests that universal school-based prevention programs may not be sufficient alone and, most importantly, that these programs are likely not reaching a sufficient threshold of at-risk children to see an impact in the incidence of CSA rates.

The school system provides an ideal implementation infrastructure for prevention programs, as evidenced by the implementation of school-based programs targeting bullying [18], substance use [19], and mental health [20]. Schools serve students across all racial, ethnic, and economic statuses and, as such, provide the highest likelihood of reaching a vast number of children at risk of CSA at one time. CSA prevention programs have also been implemented in schools, specifically elementary schools [21]. Programs offered in schools broadly aim to increase students’ knowledge and use of protective skills to prevent abuse, and several universal school-based programs have been successful in improving children’s knowledge of self-protection skills [22,23,24], including *Safe Touches*.

*Safe Touches*, developed by The New York Society for the Prevention of Cruelty to Children (NYSPCC), is an evidence-based CSA prevention program designed for students in kindergarten through third grade. The 50-min classroom-based workshop uses culturally diverse puppets, role-play scenarios (i.e., skits), and interactive discussion to teach students to identify private parts of their body, the difference between safe and not-safe touches, and the distinction between secrets and surprises [23]. Importantly, *Safe Touches* underscores that should an unsafe touch occur, it is not the child’s fault, and emphasizes the importance of telling a trusted adult. The original trial of *Safe Touches* (N = 492) conducted in New York City public schools demonstrated a significant increase in children’s CSA-related knowledge among those who received the workshop compared to those who did not, and the observed gains were highest among second graders (approximately 7–8 years old) [23,24]. A longitudinal cohort study (N = 2029) conducted across five counties in a mid-Atlantic state replicated students’ improvement of CSA-related knowledge after the *Safe Touches* workshop, and added to the evidence by demonstrating that gains were maintained at 6- and 12-months post-workshop [22,25]. Since 2007, *Safe Touches* has been implemented in the New York City public school system and has been scaled up across the mid-Atlantic state, as well as in Greece.

As designed, *Safe Touches*—like most school-based CSA prevention programs—is delivered by two trained facilitators in the classroom performing live skits with puppets to teach children the body safety concepts. The number of facilitators is solely a feature of conducting the live puppet skit. Informed by a large-scale implementation trial through which 14,235 second grade students received *Safe Touches*, a cost analysis indicated the average per-child cost was USD $43 (equating to a per-site cost of USD $154,243), mostly driven by facilitator time (e.g., salary and benefits; see [26]). By necessity, an alternative delivery modality was examined during the COVID-19 pandemic when schools were closed as a means of preventing transmission. During this unprecedented time, *Safe Touches* transitioned to virtual delivery via interactive videoconferencing (IVC) platforms, i.e., Zoom or Google Classrooms. In the virtual delivery of *Safe Touches*, the trained facilitator used IVC to lead interactive discussions supported by pre-recorded videos of the puppet-based skits. The acceptability and feasibility of the virtual delivery were examined in a pilot study [27], and this is the subject of an ongoing equivalence trial. Notably, the virtual delivery produced post-workshop CSA-related knowledge that was comparable to the in-person delivery and implicitly reduced facilitator-related costs (e.g., requiring only one facilitator for delivery) as well as travel costs (e.g., no mileage). Adopting a variety of alternative delivery approaches allows for flexibility in the continuation of implementation when school closures arise for whatever reason (e.g., weather, sick days), but may also be advantageous in bolstering the dissemination of school-based CSA prevention programs. It is unknown if the live puppet show conducted by two facilitators is essential to seeing an increase in CSA-related knowledge following the *Safe Touches* workshop.

Ubiquitously, school-based CSA prevention programs are subject to a multitude of implementation barriers, including a high rate of staff turnover, which commonly inhibits the implementation of the intervention as designed. For example, the subject of the current pilot study is a site that had a long history of implementing *Safe Touches* and was previously trained in the virtual delivery option during COVID-19. The site experienced a sudden staff change and budget cuts (e.g., being unable to invest in the staffing and training of a second facilitator). With only one trained facilitator, the site was at risk of not being able to continue its implementation. This would have left many schools in the lurch as the schools had adopted and integrated this preventive education into the curriculum [28]. So as to not fall short on the commitment to schools with whom the site had long-established relationships, the remaining one facilitator, on her own accord, continued implementation by delivering the *Safe Touches* workshop in the classroom using the pre-recorded videos resulting in a natural experiment; this is the subject of the current study.

A natural experiment occurs when an intervention is not controlled or manipulated by researchers, thus requiring alternative non-experimental design options [29]. Leveraging the aforementioned variation, or modification, in the implementation of *Safe Touches* by one facilitator using pre-recorded videos as a natural experiment, this study sought to explore the feasibility of the single-facilitator-delivered workshop. Feasibility indicates the viability of a larger randomized trial. Feasibility and acceptability were examined multimodally. First, we compared students’ self-reported CSA-related knowledge immediately after the *Safe Touches* workshop for both modalities. Then, to enrich the quantitative knowledge assessment, we assessed the acceptability of the delivery modality through teacher and counselor perceptions of the workshop as well as qualitative input from the facilitator. Expanding the toolbox of implementation (or delivery) options for school-based CSA prevention programs, such as *Safe Touches*, increases the reach and impact of these important prevention programs.

## 2. Materials and Methods

### 2.1. Participants and Procedures

Data come from a program evaluation conducted at ten sites across a mid-Atlantic state, the procedures of which were approved by the University’s Institutional Review Board. As these data were collected under the guise of program evaluation, informed consent was not required.

The current study leveraged a natural experiment at one site, with a victim service agency serving two rural counties. The site had implemented Safe Touches since 2018 with two facilitators trained in the original delivery modality (in-person delivery) as well as virtual delivery (one facilitator via IVC; see [27]). From September to mid-October 2022 the workshop was delivered as designed, when the site was unexpectedly reduced to one trained facilitator. Then, in mid-October 2022 through March 2023, implementation continued with the one-facilitator-delivered workshop.

Following the school district’s standard procedures, parents were notified their child(ren) would soon receive the Safe Touches workshop. Parents could choose to opt their children out of the workshop. Students who did not have their parent’s permission to participate (N = 45) completed an alternative supervised activity during the workshop. After the workshop, students were invited to complete a post-workshop evaluation. As this was collected as part of a program evaluation, the Institutional Review Board determined that parent permission (i.e., consent) was not required. Students who agreed completed a paper survey under the supervision of the facilitator(s) as well as the teacher and counselor. Data presented herein are extracted from these post-workshop program evaluations. Students had not previously received the Safe Touches workshop in a different grade level.

### 2.2. Safe Touches Workshop Delivery

The 50-min Safe Touches workshops were offered in two modalities: (1) two facilitators in the classroom (as designed) and (2) one facilitator in the classroom using pre-recorded puppet skit videos. For the in-person delivery, two facilitators were in the classroom to act out scenes with puppets and foster an interactive discussion (hereafter referred to as Safe Touches as designed). By contrast, the one-facilitator-led workshop (hereafter referred to as one-facilitator-led Safe Touches) was in the classroom using the pre-recorded skits and script, initially developed for the virtual delivery option [27], and engaged the students in an interactive discussion. The curriculum content and post-workshop evaluation procedures did not diverge across the two delivery modalities and all students received an activity-based booklet to take home following the workshop.

### 2.3. Measures

#### 2.3.1. CSA Questionnaire

The primary outcome was the CSA-related knowledge questionnaire, adapted from the Children’s Knowledge of Abuse Questionnaire (CKAQ; see [30]) that has been used in previous evaluations of Safe Touches [22]. The modified version includes six items related to appropriate and inappropriate touches, collected on a 3-point scale ranging from 0 (false) to 1 (in between) to 2 (true), with a higher score indicating a higher degree of knowledge [28]. For example, for the item “You have to let grown-ups touch you whether you like it or not”, students indicated whether they believed this to be false, in between (i.e., unsure), or true. Four additional items were added to provide practice with the structure of response options (i.e., “Cats are better than dogs”) and to collect data on the school environment (i.e., “I look forward to coming to school every day”, “I have good friends in my classroom”, and “My school is a happy and safe place to be”). The present analysis focused on item-level responses and ‘in-between’ responses were coded as incorrect. Each item showed an acceptable reliability using a Cronbach’s alpha score (0.60–0.70) and a moderate internal consistency of α = 0.63. The post-workshop evaluation also asked students to provide answers to two demographic questions. Age was measured as a continuous variable. Gender was a categorical variable assessed as boy and girl. These characteristics are presented descriptively.

#### 2.3.2. Qualitative Feedback

To enrich the assessment of students’ CSA-related knowledge, we collected teacher (and/or counselor) feedback on program delivery and student engagement during the workshop. The teacher’s evaluation, collected immediately following the workshop, assessed their knowledge and acceptability of the *Safe Touches* workshop where six items with 5-point Likert response options ranged from strongly disagree (1) to strongly agree (5). The first and senior authors conducted a 45-min debrief interview on Zoom with the facilitator who had delivered both modalities at the site using a semi-structured interview guide. The interview was transcribed verbatim, de-identified by the first author, and reviewed by the entire research team.

### 2.4. Analytic Approach

The student-level post-workshop program evaluation data were collected on paper and then entered by undergraduate research assistants into REDCap for data management [31]. Quantitative analyses were conducted using the Statistical Package for the Social Sciences (SPSS) software version 28.0 (IBM, Armonk, USA). Descriptive statistics were denoted as means and standard deviations (SD) for continuous variables, and numbers (*N*) and percentages (%) for categorical variables. The comparison of CSA-related knowledge item responses for the two workshop delivery modalities was assessed using an independent-samples *t*-test, where *p* < 0.05 was considered significant. The qualitative data from the interview were thematically coded and were triangulated with the quantitative student data and teacher evaluations to understand the feasibility and acceptability of the novel, one-facilitator-led delivery modality. Exemplar quotes from the facilitator are presented for context.

## 3. Results

Between September 2022 and March 2023, the *Safe Touches* workshop was implemented in 88 classrooms in 25 schools across two rural counties, reaching a total of N = 1480 second-grade students (N = 490 received *Safe Touches* as designed; N = 990 received the one-facilitator-led workshop). Participants were mostly seven years old (66%), with a mean age of 7.35 years (SD = 0.52) and range 7–9 years. Slightly more than half (51%) of the student participants self-identified as boys.

### 3.1. CSA-Related Knowledge

Overall, the means of each item were largely similar for both delivery modalities (Table 1). There were three items with a significant difference observed between modalities. A significant difference was found for item 2 (i.e., “You have to let grown-ups touch you whether you like it or not”), with a mean CSA score for students who received one-facilitator-led workshop of M = 1.44 (SD = 0.77) and a significantly higher mean difference compared to workshops delivered as designed [M = 1.35, SD = 0.80; t(1474) = 1.966, *p* < 0.05]. There was also a significant difference on item 3 (i.e., “You can trust your feelings about whether a touch is good or bad”), in which the mean score among students who received the one-facilitator-led workshop (M = 1.56, SD = 0.71) had a significantly lower mean difference compared to workshops delivered as designed [M = 1.64, SD = 0.66; t(1469) = (−2.118), *p* < 0.05]. Lastly, a significant difference in means was observed for item 8 (i.e., “You always have to keep secrets”), where the mean score for students who received the one-facilitator-led workshop (M = 1.79, SD = 0.55) had a significantly higher mean difference compared to workshops delivered as designed [M = 1.72, SD = 0.59; t(1464) = 2.381, *p* < 0.01]. The remaining items were not significant, indicating no difference in the item-level means across both modalities.

### 3.2. Teacher’s Perspective on the Safe Touches Workshop Delivery Modalities

A total of 76 teachers completed the post-workshop evaluation, of whom, 23 viewed Safe Touches as designed and 53 viewed the one-facilitator-led Safe Touches workshop in their classrooms (Table 2). Across both modalities, the majority of teachers reported that the workshop content was presented clearly (90%). Most of the teachers (80.3%) strongly agreed that the children were engaged during the workshop, with a nominally higher proportion of teachers who viewed the one-facilitator-led workshop strongly agreeing compared to teachers who viewed the workshop as designed, 87% vs. 65%, respectively. The majority of teachers (86%) indicated that the facilitator responded effectively to the children’s questions and comments, with a higher percentage who received the one-facilitator-led Safe Touches workshop compared to the workshop as designed, 91% vs. 74%, respectively. A similar proportion of teachers who viewed both modalities, 42% and 44%, indicated that following the workshop, they had a better understanding of sexual abuse prevention and body safety education. Two-thirds (67%) of all teachers indicated their intent to reinforce the sexual abuse prevention and body safety concepts with students, with a nominally higher proportion observed among teachers who viewed the one-facilitator-led workshop compared to teachers who viewed the workshop as designed, 70% vs. 61%, respectively. The majority of teachers (80%) were in full agreement that they would recommend the workshop to their colleagues, with a nominally higher endorsement among those who viewed the one-facilitator-led workshop compared to teachers who viewed the workshop as designed, 85% vs. 70%, respectively.

### 3.3. Facilitator Perspective on the Two Delivery Modalities

Consistent across modalities, the facilitator spoke about the challenge of student engagement and offered some strategies:

“Classroom management is tricky. You have to set expectations right out of the gate, let them know what you’re going to be doing. And also, I think, being repetitive in a sense of you know, the skills in every, in the knowledge that you’re presenting to them, a lot of repeating a lot of letting them know what’s okay and what’s not okay.”

When asked about the disadvantages of the two delivery modes, the facilitator commented, “I don’t know that there [are] any drawbacks. I would say that the biggest drawback of two facilitators would be travel.” Located in a rural setting and serving two contiguous counties, the facilitator explained that travel hindered the number of classrooms she could serve in a week. She elaborated that the one-facilitator-led workshops could also allow a site to send facilitators to two different schools on the same day or to do workshops in a school simultaneously—thereby increasing the potential reach.

She reported that the one-facilitator-delivery with the pre-recorded video modality “wasn’t entirely different” from the two-facilitator delivery. She noted no notable differences in the implementation of the program and no observed difference in student engagement:

“You’re still using all the same discussion language that you want with the two facilitators and the live puppet show. So all of the discussion, all of that in-between things that you’re saying and talking to the students about is all the same.”

The facilitator noted that the engagement of school personnel, specifically teachers and counselors, was largely logistically focused: “I schedule with the [counselor]… some schools will have a counselor with me all day.” During the workshop, the facilitator felt that regardless of the delivery modality, school personnel were:

“Not really engaged, but in the classroom at the same time, you know, taking me from room to room. A lot of teachers use it as time that even if they’re in the classroom, they use it as time, you know, to do some other things that might need to be done.”

School personnel engagement is especially important should a disclosure be made during a workshop. This ensures the child is connected to the appropriate resources, as needed. While the present pilot study did not implement Safe Touches virtually, in which the facilitator engages with students via IVC, the facilitator had experience in delivering the workshop in this modality during COVID-19. She described the difference in handling disclosures if she were to ‘Zoom’ into the classroom:

“There could be five disclosures missed if I’m not in the classroom doing a program like this. And I think the teacher has to be way more involved if I’m zooming into the classroom, I can’t call on students to answer a question, because I can only see three of them on the screen. And then it’s the teacher repeating, because I can’t hear.”

Her physical presence in the classroom—in both delivery modalities examined herein—increased her confidence in the ability to ascertain and execute the appropriate response to questions and disclosures.

Although the one-facilitator-delivered workshops were not planned or anticipated, the facilitator expressed her excitement about having an alternative option and continuing the implementation: “I like both… 100% [do not] prefer one way or the other. I would just do whatever was necessary to get it to the students.” The facilitator said she would absolutely deliver the Safe Touches workshop again: “As long as we had the financial capacity to do it, I would 100% do it every day of my life. I love it so much.” When asked what she liked most about general Safe Touches implementation she mentioned:

“I love that it is evidence-based. That it is exactly what schools and students need. A lot of other funding sources that some of these agencies across the state doing these funding sources have certain standards to meet. And even though this is evidence-based, it doesn’t meet the standards of some of those funding sources.”

She went on to say:

“It’s also nice that I can go in for 40 min whatever the case is, deliver the program, and it truly is something that the students enjoy. But they’re also learning a lot of knowledge and skills that they can use for the rest of their life.”

## 4. Discussion

An effective intervention is only as good as its ability to be disseminated and implemented. Universal school-based CSA prevention programs, like any number of other preventive education programs, suffer from implementation barriers including a high rate of staff turnover. Alternative implementation strategies, with a demonstrated evidence-base, can improve the likelihood of successful dissemination and implementation to achieve a public health impact. This study leveraged a natural experiment to examine the feasibility of one-facilitator-led delivery with regard to students’ CSA-related knowledge, school personnel perspectives, and facilitator input.

As a whole, there was no marked difference in post-workshop CSA-related knowledge between students who received *Safe Touches* as designed compared to those who received the one-facilitator-led workshop. This aligns with the prior pilot study comparing virtual delivery to the workshop as designed [27], suggesting that the taught knowledge and skills are transferable across delivery modalities. In the current study, three items indicated a significant difference between the Safe Touches delivered as designed and the one-facilitator-led delivery modalities, but the mean differences were marginal. Further, these small differences may be explained by other factors that were not measured, such as the reading level of second-grade students in the fall compared to spring or additional school-level characteristics. The teacher evaluation statements also showed high satisfaction with the one-facilitator-led delivery modality. In addition, the facilitator, who is an experienced *Safe Touches* facilitator, noted no substantial difference in the delivery of the two modalities. The lack of difference in knowledge, acceptability by school personnel, and enthusiasm by the provider in combination suggest the viability of a large-scale trial.

Moreover, if one-facilitator-delivery of *Safe Touches* is found to be equivalently effective to delivery as designed, a notably important consideration is the potential cost efficiency. In the prior cost analysis of *Safe Touches* [26], the major cost driver was person time (i.e., salary and benefits) and travel, especially in a rural or geographically distant setting. An affordable and scalable implementation infrastructure expands the reach of universal school-based preventive education programs. This has implications for sites with high rates of turnover, but also with limited budgets and a high demand.

Though teacher evaluations noted the engagement of students during the one-facilitator-led workshops, the facilitator commented on the challenge of managing the classroom on her own. In either delivery modality, ensuring these workshops run smoothly without disruptive behavior from students compromising the delivery of instruction requires skills, patience, boundaries, and instinct. Future implementation research with new facilitators may consider focusing on classroom management in terms of training. Expert classroom management is particularly relevant when a student makes a disclosure or concerning statement requiring further conversation. Over the course of the implementation period, two disclosures were reported. It happened that both disclosures occurred during the two-facilitator delivery modality; however, the implications of this should be interpreted with caution. It is not known whether disclosures were made to school personnel following either delivery modality. That disclosures occurred at all reinforces the prior literature indicating that these workshops provide a safe place for students.

Findings from the current study, though promising, should be evaluated with consideration of some limitations. The study did not include a pre-workshop assessment or a control group. This limits our capacity to determine the level of knowledge gained. Related to this, the study was limited to post-workshop program evaluation surveys. It is unclear if the retention of knowledge would be replicated over the long term, up to 12-months post-workshop, as has been demonstrated with the workshop delivered as designed [22]. It remains unclear if students are able to retain the knowledge over a longer period of time as they move into middle school and experience situations that are developmentally significant for sexual development (e.g., at the onset of puberty). Future research on either delivery modality should consider attempts to follow participants over a longer period to ascertain whether they can retain knowledge throughout multiple developmental phases and contexts, as well as whether the *Safe Touches* workshop might result in the use of protective behaviors over time.

## 5. Conclusions

While school-based CSA prevention programs are the prevailing prevention strategy in the U.S., we would be remiss if we did not acknowledge the need for a more comprehensive approach to prevention. The burden to protect themselves should not fall on the shoulders of children alone. Adopting a public health approach to the prevention of CSA necessitates a multi-level approach that includes not only children, but adults and parents. Assini-Meytin and colleagues [31] also suggest that to realize a true reduction in the rates of CSA, there must also be a focus on perpetrators. Strategies to support the dissemination and implementation of prevention programs targeting a wide array of players in prevention are critical to the realization of a public health impact.

This study leveraged a natural experiment to explore an ad hoc modification to the implementation of an evidence-based intervention to fit a new context. This is a common occurrence in the implementation of interventions, and model developers must explore the effectiveness of alternative delivery modalities to meet the needs of the varied implementation contexts. Moreover, the availability of different formats in delivering evidence-based, universal school-based CSA prevention programs is essential to reach more students. In a time of constrained resources, alternative implementation strategies become paramount. With a menu of effective delivery modalities, universal school-based CSA prevention programs, such as *Safe Touches,* may increase the adoption of the program as well as the number of children served. An unavoidable constraint, however, concerns the available resources for school-based programs, as there is no federal legislation requiring the provision of these prevention education programs for elementary-age students. Federal or state funds supporting preventative education programs should encourage the empirical examination of alternative delivery modes so as to maximize the public health impact.

## Figures and Tables

**Table 1:** Comparison of second grade students (N = 1480) item-level CSA-related knowledge immediately post-workshop.

CSA Questions	Safe Touches as Designed				One-Facilitator-Led Safe Touches				Δ Safe Touches as Designed–One-Facilitator-Led Safe Touches *t* *
	(N = 490)				(N = 990)				
	False	In Between	True	Mean (SD)	False	In Between	True	Mean (SD)	
1. Cats are better than dogs.	205	177	108	0.80 (0.78)	397	416	176	0.78 (0.73)	−0.607
2. You have to let grown-ups touch you whether you like it or not. ^a^	271	117	99	1.35 (0.80)	601	221	167	1.44 (0.77)	1.966 *
3. You can trust your feelings about whether a touch is good or bad.	50	73	363	1.64 (0.66)	128	173	684	1.56 (0.71)	−2.118 *
4. I look forward to coming to school every day.	58	139	291	1.48 (0.70)	144	264	579	1.44 (0.73)	−0.934
5. It’s OK to say “no” and move away if someone touches you in a way you don’t like.	57	42	383	1.68 (0.68)	153	60	771	1.63 (0.74)	−1.247
6. A pat on the back from a teacher you like after you have done a good job at school is a safe touch.	28	58	400	1.77 (0.54)	47	107	834	1.80 (0.51)	1.056
7. I have good friends in my classroom.	42	69	374	1.68 (0.62)	50	223	707	1.67 (0.57)	−0.433
8. You always have to keep secrets. ^a^	379	66	35	1.72 (0.59)	848	72	66	1.79 (0.55)	2.381 **
9. Someone you know, even a relative, might want to touch your private parts in a way that feels confusing.	123	146	217	1.19 (0.82)	223	291	471	1.25 (0.80)	1.307
10. My school is a happy and safe place to be.	26	89	371	1.71 (0.56)	53	198	732	1.69 (0.57)	−0.611

Note. CSA = child sexual abuse; SD = standard deviation; Safe Touches as designed = two facilitators live in the classroom; one-facilitator-led Safe Touches = one facilitator in the classroom fostering interactive discussion around the viewing of pre-recorded puppet-based skits. ^a^ Items 2 and 8 were reverse coded for the mean score calculation; higher scores indicate a greater level of knowledge. * *p* < 0.05; ** *p* < 0.01.

**Table 2:** Frequency of teachers’ perspectives on Safe Touches workshop delivery modalities (N = 76).

Variables	Overall (N = 76)	Safe Touches as Designed (N = 23)	One Facilitator-Led Safe Touches (*r* = 53)
**The workshop content was presented clearly.**			
Strongly agree	68 (89.5%)	17 (73.9%)	51 (96.2%)
Agree	8 (10.5%)	6 (26.1%)	2 (3.8%)
Neutral	0 (0.0%)	0 (0.0%)	0 (0.0%)
**The children were engaged during the workshop.**			
Strongly agree	61 (80.3%)	15 (65.2%)	46 (86.8%)
Agree	14 (18.4%)	7 (30.4%)	7 (13.2%)
Neutral	1 (1.3%)	1 (4.3%)	0 (0.0%)
**The facilitators responded effectively to the children’s questions and comments.**			
Strongly agree	65 (85.5%)	17 (73.9%)	48 (90.6%)
Agree	10 (13.2%)	6 (26.1%)	4 (7.5%)
Neutral	1 (1.3%)	0 (0.0%)	1 (1.9%)
**My knowledge about sexual abuse prevention and body safety education has increased as a result of this workshop.**			
Strongly agree	32 (42.1%)	10 (43.5%)	22 (41.5%)
Agree	25 (39.9%)	8 (34.8%)	17 (32.1%)
Neutral	19 (25.0%)	5 (21.7%)	14 (26.4%)
**I will reinforce the sexual abuse prevention and body safety concepts taught in today’s workshop with these children.**			
Strongly agree	51 (67.1%)	14 (60.9%)	37 (69.8%)
Agree	21 (27.6%)	7 (30.4%)	14 (26.4%)
Neutral	4 (5.3%)	2 (8.7%)	2 (3.8%)
**I would recommend the Safe Touches workshop to my colleagues.**			
Strongly agree	61 (80.3%)	16 (69.6%)	45 (84.9%)
Agree	13 (17.1%)	5 (21.7%)	8 (15.1%)
Neutral	2 (2.6%)	2 (8.7%)	0 (0.0%)

## Data Availability

The raw data supporting the conclusions of this article will be made available by the authors on request.
